# VATS right posterior segmentectomy with anomalous bronchi and pulmonary vessels: a case report and literature review

**DOI:** 10.1186/s13019-021-01420-2

**Published:** 2021-03-29

**Authors:** Jianbin Zhang, Yilv Zhu, Hongwei Li, Caihua Yu, Weiwei Min

**Affiliations:** 1 Department of Thoracic Surgery, Huzhou Central Hospital, Affiliated Central Hospital of HuZhou University, 1558 Third Ring North Road, Huzhou, 313000 Zhejiang China; 2 Department of Radiology, Huzhou Central Hospital, Affiliated Central Hospital of HuZhou University, Huzhou, 313000 Zhejiang China

**Keywords:** Video-assisted thoracoscopic surgery (VATS), Three-dimensional computed tomography bronchography and angiography (3D-CTBA), Segmentectomy

## Abstract

**Background:**

Anatomic variation may increase the difficulty and risk of anatomic segmentectomy. The preoperative three-dimensional computed tomography bronchography and angiography (3D-CTBA) can provide a detailed model of the segmental structure, and contribute to precise and safe segmentectomy.

**Case presentation:**

This is a case of anomalous bronchi and pulmonary vessels in the right upper posterior segment (RS^2^). Under the guidance of 3D-CTBA, anatomic RS^2^ segmentectomy was performed accurately and safely. The postoperative condition was uneventful.

**Conclusions:**

This rare case highlights the importance of 3D-CTBA to guild accurate segmentectomy with anatomic variation.

## Background

With the popularization of chest computed tomography (CT), the discovery of small pulmonary nodules is increasingly common, which also promotes the progress of segmentectomy [1, 2]. As reported in the previous cases, the anatomic structure of the pulmonary segment is sometimes variable [3–5]. The public’s attention is now shifting to how to achieve an accurate anatomic segmentectomy. The development and application of various 3D imaging softwares have given a strong technical support in terms of the successful resection [6]. Preoperative reconstruction of the segment can clearly show the anatomic structure and judge whether there are variations, so as to make an accurate surgical plan. In this case, a 34-year-old female patient experienced a ground-glass nodule (GGN) at the right upper posterior segment (RS^2^). The preoperative three-dimensional CT bronchography and angiography (3D-CTBA) revealed multiple anatomic variations. Owing to the detailed planning of the operation, video-assisted thoracoscopic surgery (VATS) RS^2^ segmentectomy was performed successfully.

## Case presentation

**Fig. 1:**
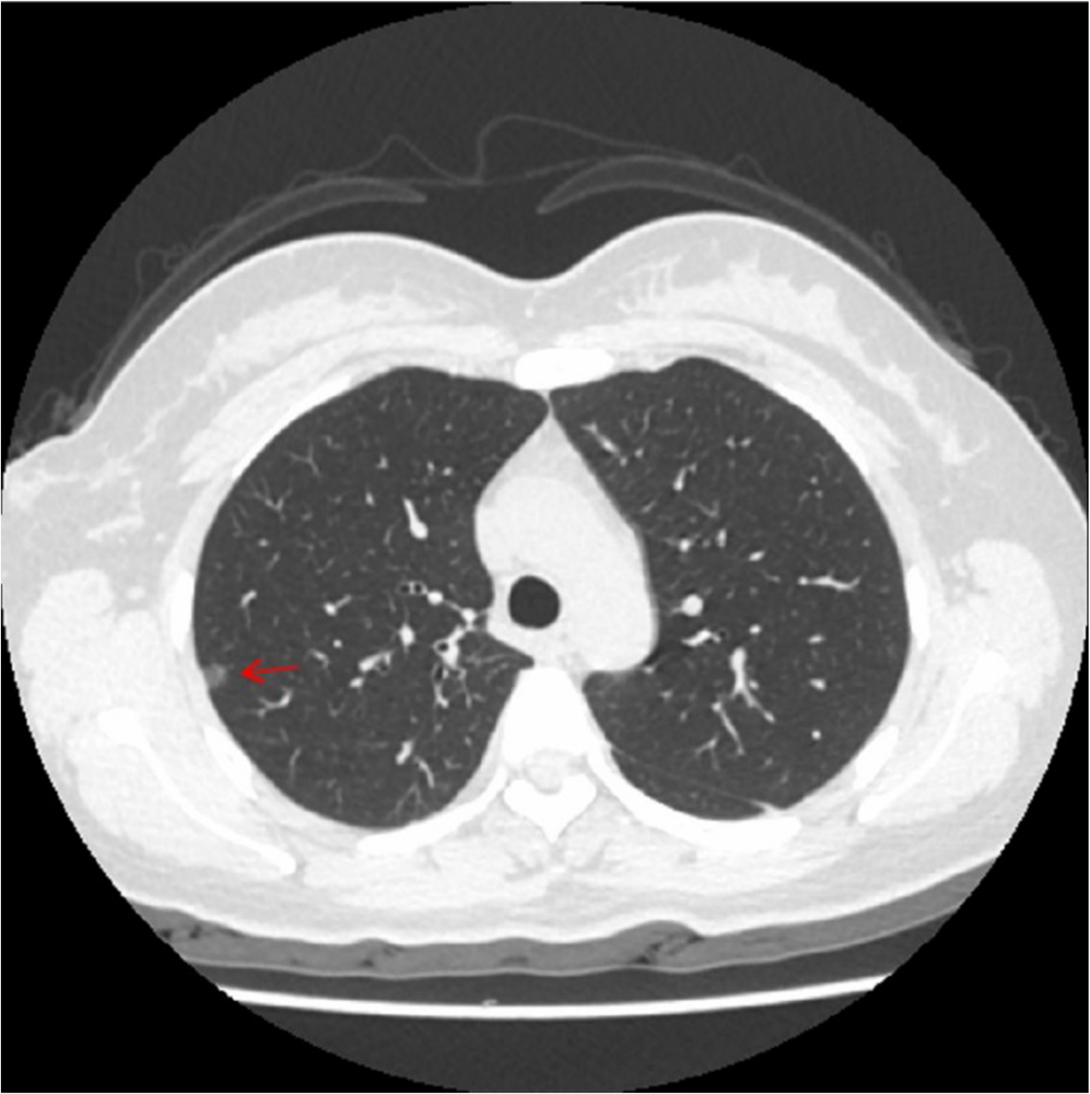
The preoperative computed tomography scanning. A 7-mm ground-glass nodule was identified at the posterior segment of the right upper lobe (arrow), the CT value was -400HU

**Fig. 2:**
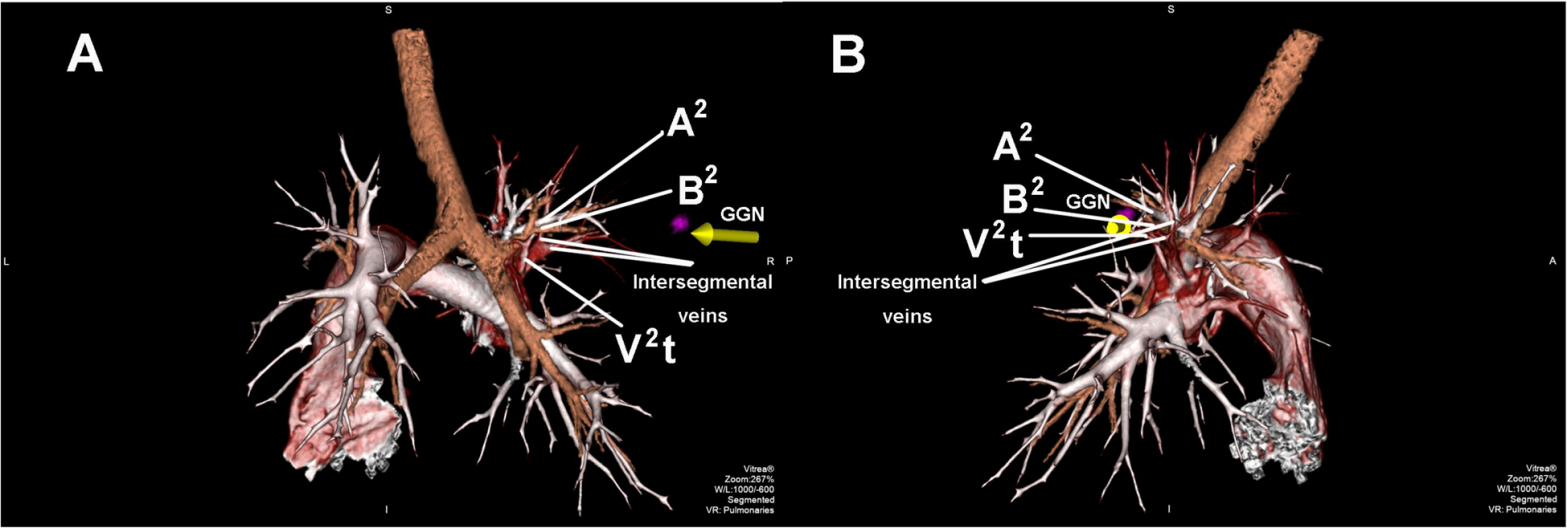
The three-dimensional computed tomography bronchography and angiography. The apical subsegmental bronchi(B^1^a and B^1^b) originated from the posterior segmental bronchus (B^2^) and the anterior segmental bronchus (B^3)^ respectively; The right upper pulmonary artery shared trunk without posterior ascending artery (Asc.A^2^); The right upper pulmonary vein had no central vein, with only one posterior intrasegmental vein(V^2^t), the other two veins pointed to the intersegmental plane respectively. **a** Posterior view; **b** Lateral view

**Fig. 3:**
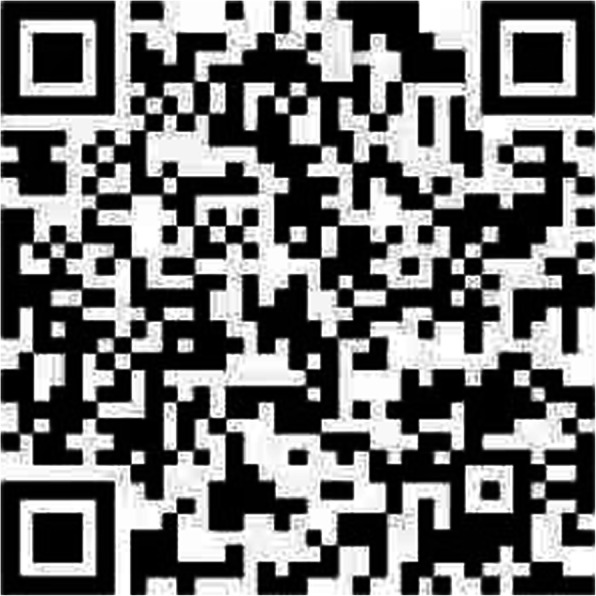
The QR code of Video. VATS right posterior segmentectomy and lymph node sampling

**Fig. 4:**
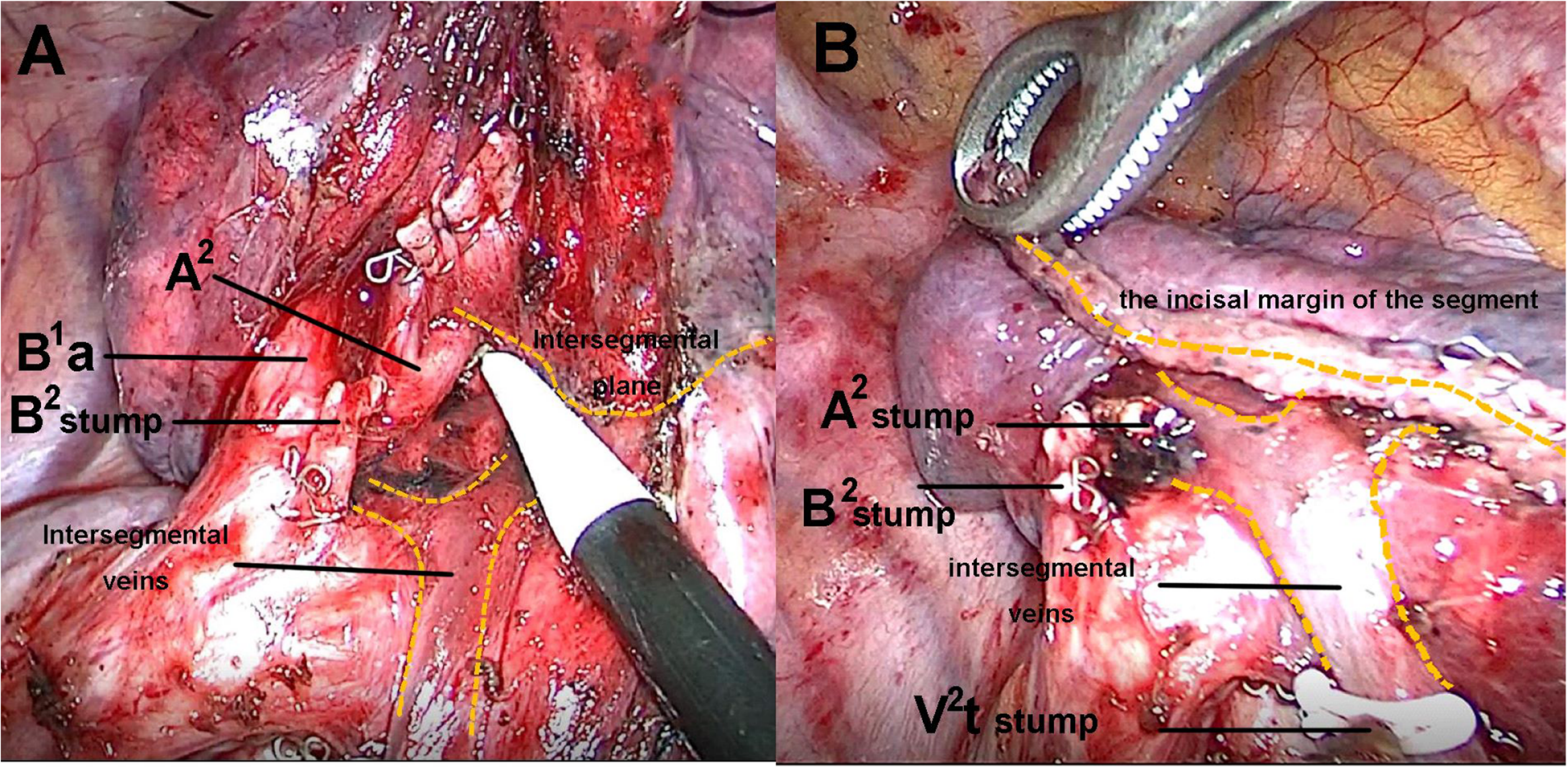
The intraoperative view of the posterior segment of the right upper lobe. **a** Exposed the posterior segmental artery after releasing the segmental hilum sufficiently; **b** The structure of lung segmental hilum after resection

**Fig. 5:**
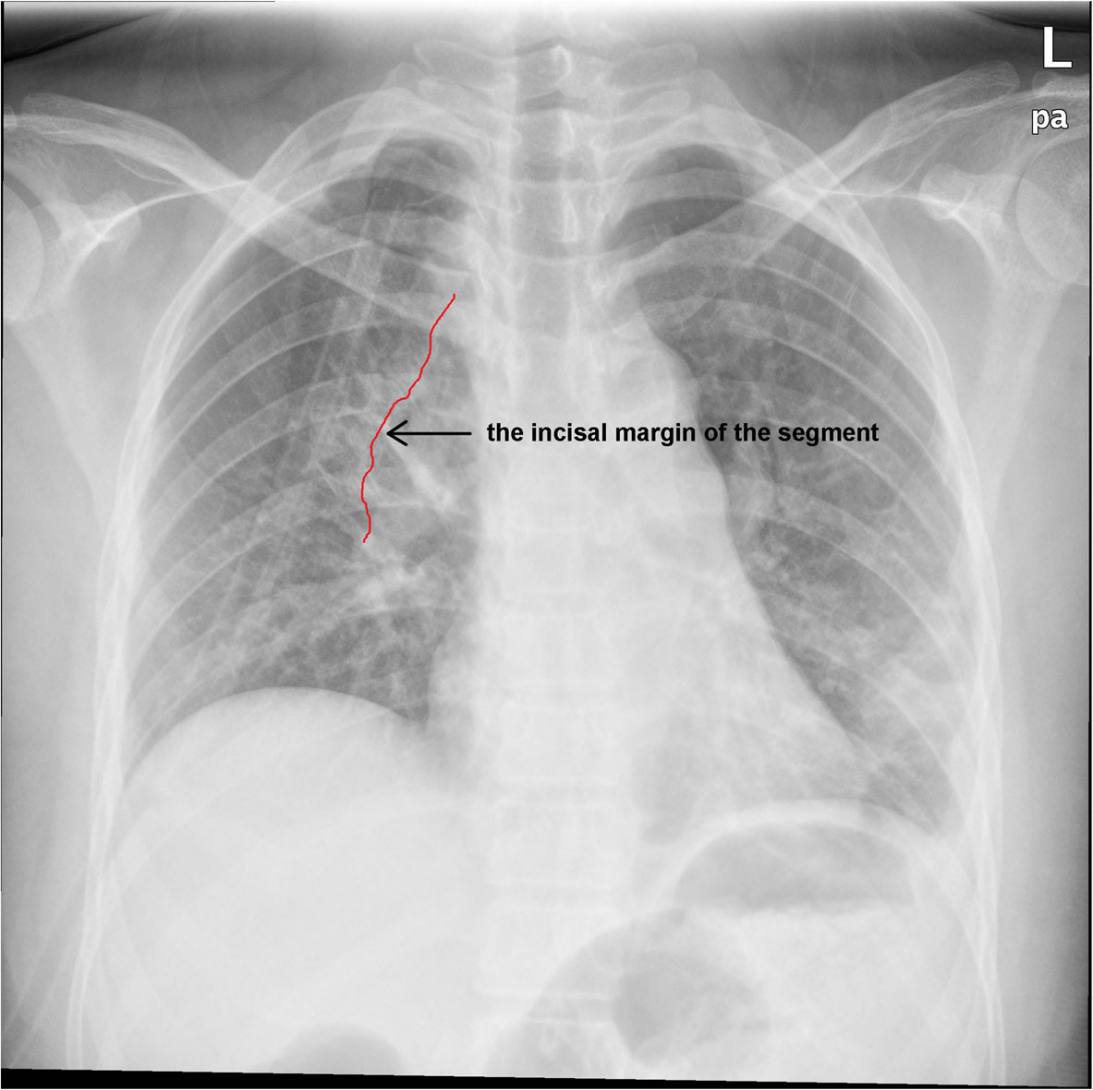
The chest radiograph on the 2nd postoperative day. The incisal margin of the segment displayed no obvious exudation (arrow)

A 34-year-old female was admitted to us with a GGN in RS^2^, which was discovered by chest CT during a health checkup 1 year ago. A review of CT indicated that the subpleural GGN was slightly larger than before, with a diameter of 7 mm and a CT value of -400HU (Fig. 1). She had no positive signs, no history of smoking, no history of malignant tumors, and no family history of lung cancer. Preoperative 3D-CTBA revealed multiple variations in the right upper lobe: (1) The apical subsegmental bronchi(B^1^a and B^1^b) originated from the posterior segmental bronchus (B^2^) and the anterior segmental bronchus (B^3^), respectively. (2) The right upper pulmonary arteries shared a trunk without a posterior ascending artery (Asc.A^2^). (3) The right upper lobe had no central vein, with only 1 posterior intrasegmental vein (V^2^t); the other 2 veins pointed to intersegmental plane respectively without another posterior intrasegmental vein (V^2^b) (Fig. 2). VATS anatomic RS^2^ segmentectomy and lymph node sampling were conducted accurately With the guidance of 3D-CTBA (Fig. 3); all anatomic variations were successfully detected during the operation (Fig. 4). Minimally invasive adenocarcinoma (MIA) was the fast-frozen pathology. The chest radiograph illustrated that the right lung was completely redilated, and on the second postoperative day, the incisal margin of the segment displayed no obvious exudation (Fig. 5). The drainage tube was removed on the second postoperative day, and the patient was discharged on the third postoperative day. The postoperative pathology revealed MIA with negative surrounding lymph nodes.

## Video description

Intravenous general anesthesia combined with double-lumen endotracheal intubation and contralateral one-lung ventilation were performed. The observation port was made in the seventh intercostal space of the posterior axillary line, and the operation port in the fourth intercostal space of the anterior axillary line. The surgical procedure was as follows: (1) Thoracoscopic exploration revealed a subpleural nodule at the RS^2^. (2) The parietal pleura was sectioned by harmonic, and then the Nos.11 lymph nodes were removed. (3) The V^2^t was dissociated and pulled by silk, and the Nos.12 and 13 lymph nodes around the bronchus were completely removed. (4) After ligating the V^2^t, the B^2^ was dissected out and transected with a thick tissular (blue cartridge) 45-mm long endostapler (EC45A, JJMC, USA). (5) According to the preoperative 3D-CTBA, the other 2 veins were judged as the intersegmental veins; hence, they were all preserved. (6) We reventilated the right lung with pure oxygen because the posterior segmental artery was very difficult to ligate; when the intersegmental plane was clear after 18 min, the segmental hilum was released sufficiently to expose the posterior segmental artery, and then the artery was ligated safely. (7) The intersegmental plane was divided along the inflation-deflation line using the endostaplers; thus, the posterior segment was removed successfully. A sterile glove was used to collect the specimen. Bleeding and air leakage were not observed after the pleural injection of inflation. No. 24 chest drainage tube was placed in the observation port. The incision was sutured after the right lung was completely dilated. The operative time was 75 min, and intraoperative blood loss was 50 mL.

## Discussion and conclusions

In recent years, anatomic segmentectomy for early lung cancer is one of the biggest hotspots in thoracic surgery. It can not only completely resect the tumor but also preserve the normal lung tissue to the maximum extent [7]. The distribution of bronchus, artery, and vein in the pulmonary segment exists variations in some patients. The key to the implementation of this operation is to accurately grasp the anatomic structure of the target segment. The 3D-CTBA can provide a precise anatomic structure, identify the intrasegmental and intersegmental veins from different views [8]. Therefore, some complicated pulmonary segmentectomy should be performed under the guidance of 3D-CTBA [9, 10].

The efficacy of 3D reconstruction for thoracic surgery has been previously described [11], Kimihiro et al. [12] first reported the application of 3D-CTBA in VATS segmentectomy. At present, the main softwares for 3D reconstruction of segment include IQQA, DeepInsight, etc. [13, 14]. All the reconstructive softwares can offer precise anatomic structure of pulmonary segments, but sometimes, it is difficult for surgeons to skillfully use them. In our institution, with the assistance of the radiologists, we adopt the CT pulmonary angiography based technology to reconstruct the anatomic structure of each patient who undergo segmentectomy. It shows the precise structure of bronchi, arteries and veins in a natural surgical field view.

In all kinds of segmental resection, RS^2^ segmentectomy is a common procedure, but anatomic variations may increase its difficulty and risk [15]. Xinfeng et al. [16] reported a tracheal bronchus and a variable central vein entering the left atrium dorsal to the right pulmonary artery trunk in a patient who underwent VATS RS^2^ segmentectomy. Tadashi et al. [17] reported anatomic variations in bronchi and pulmonary vessels in a patient who underwent thoracoscopic RS^2^ segmentectomy. However, to the best of our knowledge, bronchial variation associated with variant pulmonary vessels has rarely been reported. The normal anatomic structure of RS^2^ consists of B^2^, Asc.A^2^, the recurrent artery (Rec.A^2^), intrasegmental veins (V^2^t and V^2^b) and intersegmental veins (V^2^a and V^2^c), but in this case, the 3D-CTBA revealed multiple variations: (1) The bronchopulmonary trees of the right upper lobe were divided into (B^2^ + B^1^a) and (B^3^ + B^1^b); the (B^2^ + B^1^a) variation might have been mistaken for B^2^ without the guidance of 3D-CTBA. (2) There was only 1 intrasegmental vein (V^2^t) without another intrasegmental vein (V^2^b); intersegmental veins might have been transected as intrasegmental veins without the preoperative 3D-CTBA. (3) The posterior segmental arteries originated from the superior trunk without Asc.A^2^. They were very difficult to expose; therefore, we released the hilum of segment first to expose the arteries sufficiently, and then the artery was safely ligated. This novel strategy has rarely been reported. In conclusion, we present a successful strategy for VATS RS^2^ segmentectomy with multiple anatomic variations.

## Data Availability

All data generated or analysed during this study are included in this article.

## Abbreviations

3D-CTBA: Three-dimensional computed tomography bronchography and angiography
RS^2^: Right upper posterior segment
VATS: Video-assisted thoracoscopic surgery
CT: Computed tomography
GGN: Ground-glass nodule
MIA: Minimally invasive adenocarcinoma
B^1^: Apical segmental bronchus
B^2^: Posterior segmental bronchus
B^3^: Anterior segmental bronchus
Asc.A^2^: Ascending artery
Rec.A^2^: Recurrent artery.
